# Curcumin in Ophthalmology: Mechanisms, Challenges, and Emerging Opportunities

**DOI:** 10.3390/molecules30030457

**Published:** 2025-01-21

**Authors:** Adriana Ribeiro, Daniele Oliveira, Helena Cabral-Marques

**Affiliations:** 1 Research Institute for Medicines (iMed.ULisboa), Faculty of Pharmacy, Universidade de Lisboa, 1649-003 Lisboa, Portugal; 2 Ophthalmologist, Centro Hospitalar de Setúbal and Hospital da Luz Setúbal, 2900-722 Setúbal, Portugal; daniele.oliveira@edu.ulisboa.pt

**Keywords:** curcumin, ocular diseases, drug delivery systems, bioavailability, nanoparticles, cyclodextrin complexes, anti-inflammatory, neuroprotective properties, posterior segment diseases, ophthalmic therapies

## Abstract

Ocular diseases affecting the anterior and posterior segments of the eye are major causes of global vision impairment. Curcumin, a natural polyphenol, exhibits anti-inflammatory, antioxidant, antibacterial, and neuroprotective properties, making it a promising candidate for ocular therapy. However, its clinical use is hindered by low aqueous solubility, poor bioavailability, and rapid systemic elimination. This review comprehensively highlights advances in curcumin delivery systems aimed at overcoming these challenges. Emerging platforms, including proniosomal gels, transferosomes, and cyclodextrin complexes, have improved solubility, permeability, and ocular retention. Nanoparticle-based carriers, such as hybrid hydrogels and biodegradable nanoparticles, enable sustained release and targeted delivery, supporting treatments for posterior segment diseases like diabetic retinopathy and age-related macular degeneration. For anterior segment conditions, including keratitis and dry eye syndrome, cyclodextrin-based complexes and mucoadhesive systems enhance corneal permeability and drug retention. Mechanistically, curcumin modulates key pathways, such as NF-κB and TLR4, reducing oxidative stress, angiogenesis, and apoptosis. Emerging strategies like photodynamic therapy and neuroprotective approaches broaden their application to eyelid conditions and neuroinflammatory ocular diseases. These advancements address curcumin’s pharmacokinetic limitations, supporting its clinical translation into ophthalmic therapies. This work underscores curcumin’s potential in ocular disease management and advocates clinical trials to validate its safety, efficacy, and therapeutic relevance.

## 1. Introduction

Ocular diseases, encompassing retinal and corneal disorders alongside ocular surface conditions, such as eyelid pathologies, significantly contribute to the global burden of visual impairment [1,2,3,4]. These conditions pose significant challenges in prevention and treatment. For instance, diabetic retinopathy (DR) affected over 100 million people globally in 2020, with projections surpassing 160 million by 2045 [5]. Similarly, glaucoma, a leading cause of irreversible blindness, is estimated to impact 111.8 million people aged 40–80 worldwide by 2040 [6]. Dry eye syndrome impacts a significant portion of the population, with prevalence rates varying widely between 5% and 50%, underscoring the growing burden of age-related ocular conditions, which are expected to increase with the aging population, projected to double to 2.1 billion by 2050 [7,8].

Moreover, lifestyle factors, such as unhealthy eating habits, smoking, and the frequent use of digital devices, have intensified these challenges. Worryingly, projections from the Global Burden of Disease Study suggest that by 2050, approximately 474 million people may experience moderate to severe visual impairments, with 61 million potentially losing their sight entirely [9].

In recent years, considering these obstacles, there has been growing interest in curcumin as a possible therapeutic agent in managing ocular diseases. Curcumin (C_21_H_20_O_6_), a lipophilic polyphenol derived from the dried rhizome of *Curcuma longa* L. and related species, has gained significant attention due to its extensive pharmacological properties, including anti-inflammatory, antioxidant, antimicrobial, and antitumor activities [10,11,12]. Alongside its primary forms—demethoxycurcumin and bis-demethoxycurcumin—turmeric contains over 50 additional curcuminoids, including bisabocurcumin, curcumalongin, cyclocurcumin, and terpecurcumin, as well as volatile oils and resins. These compounds broaden turmeric’s pharmacological profile, offering synergistic effects that enhance its therapeutic versatility and reinforce its global use both as a culinary spice and as a source of health benefits [13].

Curcumin’s molecular structure, comprising two o-methoxy phenolic aromatic rings linked by a seven-carbon α, β-unsaturated β-diketone chain, underpins its pleiotropic effects. Its properties—anti-inflammatory, antioxidant, antibacterial, anti-angiogenic, and anti-apoptotic—show promise in ophthalmology [14,15]. Research indicates its potential for treating corneal and retinal neovascularization, inhibiting lens epithelial cell proliferation, and modulating retinal pigment epithelium-related pathways, making it a valuable candidate for managing inflammatory and degenerative ocular diseases [16,17]. Topical formulations, such as hydrogels, creams, and nanocarrier systems, have been developed to enhance their physicochemical properties, including solubility, permeability, and stability. These innovations protect curcumin from degradation and enable sustained release, proving effective in treating dermatological conditions, such as psoriasis, acne, and atopic dermatitis, due to its anti-inflammatory, wound-healing, and antioxidant properties. This success in dermatology has spurred interest in its application for ocular treatments due to similar physicochemical barriers [18,19].

Recognized by the FDA as ‘Generally Recognized as Safe’ (GRAS) for human consumption, curcumin demonstrates significant therapeutic potential [20]. Clinical trials have confirmed their excellent safety, tolerability, and efficacy, even at high oral doses ranging from 4 to 8 g per day and doses up to 12 g per day for curcuminoid formulations containing 95% curcumin, bisdemethoxycurcumin, and demethoxycurcumin [20,21]. However, its clinical application is limited by critical pharmacokinetic challenges, including poor aqueous solubility, light sensitivity, low bioavailability, limited absorption, and rapid systemic metabolism and elimination. These factors complicate consistent therapeutic outcomes and pharmacological interpretations, particularly given curcumin’s classification as a PAIN (pan-assay interference compound) and an IMP (invalid metabolic panacea), which highlights its complex bioactivity. Its degradation of products and fluorescence further complicate pharmacological evaluations [22,23]. These limitations not only hinder consistent therapeutic outcomes but also complicate the identification of the actual bioactive species responsible for its effects.

In addition, the metabolism of curcumin and its interaction with the intestinal microbiota play a crucial role in determining its bioavailability and therapeutic efficacy. After oral administration, curcumin exhibits poor solubility and limited gastrointestinal absorption. The absorbed fraction undergoes rapid metabolism in the liver and intestine via reduction (yielding dihydrocurcumin and tetrahydrocurcumin) and conjugation (forming glucuronides and sulfates), leading to its swift elimination. The intestinal microbiota further converts curcumin into more stable and, in some cases, bioactive metabolites. Certain bacterial genera, such as *Bifidobacterium* and *Lactobacillus*, promote its reduction and demethylation, potentially enhancing biological activity. Conversely, curcumin modulates the gut microbiota, fostering beneficial species while inhibiting pathogens. These interactions have significant implications for curcumin’s therapeutic applications in ocular diseases, especially given the distinct metabolic pathways associated with oral and topical administration [24].

Additionally, the wide range of commercially available formulations—ranging from turmeric powder to curcuminoid-enriched products and purified curcumin—adds complexity, raising concerns about reproducibility and efficacy in clinical trials [2,25,26,27,28]. Despite these challenges, curcumin’s diverse therapeutic potential emphasizes the need for innovative delivery systems.

Nanocarrier technologies, particularly vesicular systems, such as liposomes and proniosomes, have addressed some of these challenges by improving curcumin’s bioavailability, solubility, and stability [29]. These systems encapsulate curcumin within surfactant vesicles, protecting it from enzymatic degradation and extending its therapeutic presence on ocular surfaces. Moreover, the sustained drug delivery provided by these systems reduces systemic side effects while targeting disease-specific sites [30].

In ocular inflammation, curcumin has demonstrated efficacy in reducing complications, such as corneal opacity, cataract formation, and retinal detachment [3]. It also holds promise as a prophylactic agent in proliferative vitreoretinopathy (PVR), with studies reporting reduced rates of retinal detachment following surgery [31]. Furthermore, curcumin demonstrates therapeutic benefits in DR by modulating hyperglycemia-induced endothelial dysfunction [32].

Topical drug delivery systems, such as eye drops, i.e., aqueous solutions and suspensions, and oil-based formulations, remain widely used for ocular treatment. These formulations are intended for direct application to the ocular surface, typically in the form of drops. However, they often face significant limitations, including excessive tear production, rapid drainage, and systemic absorption, leading to inefficient drug distribution and the loss of over 95% of the administered dose [33].

This review explores curcumin’s therapeutic potential in ophthalmology, focusing on its molecular mechanisms, challenges in clinical application, and advanced strategies for optimized delivery. By addressing these barriers, curcumin could transform ocular disease management, highlighting the need for robust randomized trials to confirm its safety and efficacy.

## 2. Therapeutic Applications of Curcumin in Ophthalmology

Delivering medications to the eye effectively remains a significant challenge due to its distinct pharmacokinetic and pharmacodynamic environment. The eye’s natural defense mechanisms—such as tear production, blinking, and the intricate clearance processes on the ocular surface—serve to protect it but simultaneously hinder drug retention and absorption. These barriers, coupled with the anatomical complexity of anterior and posterior segments, result in low bioavailability for many conventional therapies [34,35]. Frequent application of traditional eye drops is often necessary to achieve therapeutic outcomes, but this practice can inadvertently lead to systemic side effects through absorption via the nasolacrimal pathway [30,36].

**Figure 1:**
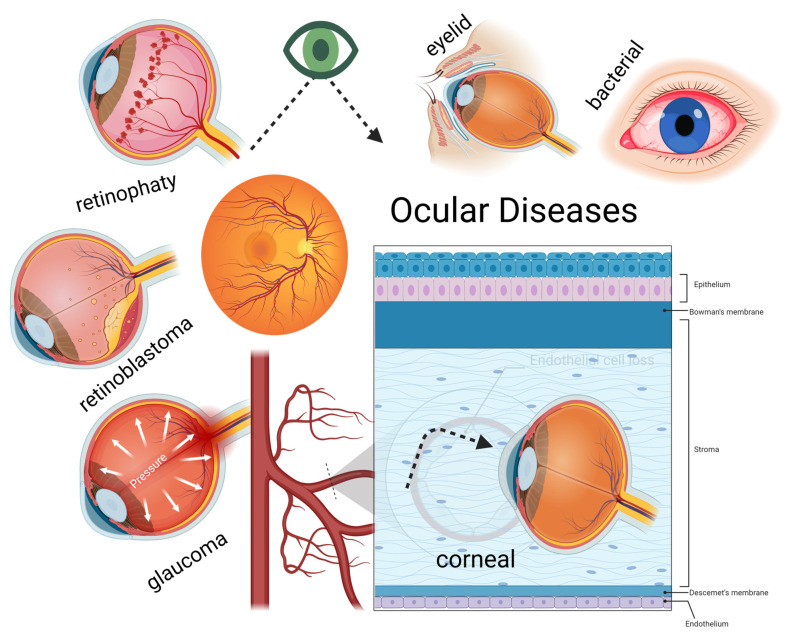
Major ocular diseases in human eye.

Recent studies suggest that curcumin can be applied to various ophthalmic conditions, offering significant therapeutic potential for a wide range of ocular diseases and addressing many limitations of conventional approaches (Figure 1). Curcumin’s antimicrobial and immunomodulatory properties make it particularly effective in targeting the complex interplay of infection, inflammation, and oxidative stress underlying many ocular pathologies. It directly disrupts bacterial cell walls and inhibits enzymatic processes critical for bacterial survival while also downregulating pro-inflammatory cytokines and mitigating oxidative stress. These dual antibacterial and anti-inflammatory actions position curcumin as a versatile therapeutic agent, particularly when integrated into advanced drug delivery systems [37,38].

As shown in Table 1, the development of advanced drug delivery systems, including in situ gels, nanostructured lipid carriers, and hydrogels, has emerged as a promising strategy to enhance the solubility, stability, and ocular bioavailability of curcumin.

An in vitro study, supported by ex vivo assays using rabbit corneas, showed that polyethylene glycol-distearoylphosphatidylethanolamine (PEG-DSPE)/Solutol HS 15 mixed micelle-based in situ gels improve corneal penetration, ocular retention, and stability. This system also supports sustained drug release, reduces dosing frequency, and avoids ocular irritation, offering a promising alternative to conventional eye drops [39].

Thiolated chitosan-coated nanostructured lipid carriers, characterized in vitro and further evaluated in vivo using animal models, enhance corneal contact through covalent bonding with mucus glycoproteins. This interaction ensures sustained release over 72 h without irritation, improving ocular distribution and therapeutic efficacy [40].

Hydroxypropyl methylcellulose methacrylate hydrogels, tested in vivo, demonstrated strong bioadhesion and controlled curcumin release, contributing to the reduction of oxidative damage in trabecular meshwork cells. This effect helps mitigate the inflammatory and apoptotic processes that are key in glaucoma progression, confirming the safety and potential of this formulation for controlled ocular drug delivery [41].

These studies highlight the potential of these technologies to improve ocular retention, reduce dosing frequency, and minimize systemic side effects. Such advancements enable sustained drug release, improve therapeutic outcomes, and alleviate the burden of frequent applications associated with traditional formulations in ocular diseases.

To better understand the impact of delivery methods on curcumin’s bioavailability and therapeutic potential, Table 1 provides a comparison of different routes of administration, including topical ocular systems, oral administration (including trial clinical phase I), and parenteral routes.

Ocular drug delivery systems offer a distinct advantage by bypassing the first-pass metabolism typical of oral administration and directly targeting the eye.

These formulations improve curcumin’s retention, permeability, and controlled release, leading to enhanced therapeutic efficacy when compared to oral or intravenous routes. Additionally, ocular systems minimize systemic exposure, reducing the likelihood of side effects and providing a more focused and controlled therapeutic approach.

**Table 1:** Curcumin’s ocular therapeutic potential.

Route of Administration	Formulation/Technology	Key Findings	Bioavailability/Retention	Refs.
Topical	Curcumin-loaded in situ gel (eye drops)	Enhanced solubility and corneal penetration	Increased bioavailability (not quantified)	[39]
Topical	Chitosan–curcumin–nano-lipid carriers (eye drops)	Improved permeability and ocular retention	6.4–18.8-fold increase	[40]
Topical	Hydroxypropyl methylcellulose hydrogels	Sustained release, reduced oxidative damage	Sustained release over 6–8 h	[41]
Oral	Conventional oral curcumin (3600–8000 mg/day)	Low bioavailability, rapid metabolism	Poor systemic bioavailability	[42,43,44]
Oral	Theracurmin(enhanced oral curcumin)	Increased systemic exposure (18.4–20.5 times higher)	Higher plasma concentration	[45]
Intravenous	Intravenous administration in rats (10 mg/kg)	Rapid plasma peak concentration (0.36 µg/mL)	High but short-lived bioavailability	[28]
Intramuscular	Intramuscular injection in rats(50 mg/kg)	Sustained plasma concentration	Bioavailability ~30%	[46]
Intraperitoneal	Intraperitoneal administration in rats (20 mg/kg)	Higher bioavailability	Bioavailability 35.07%	[47]

**Figure 2:**
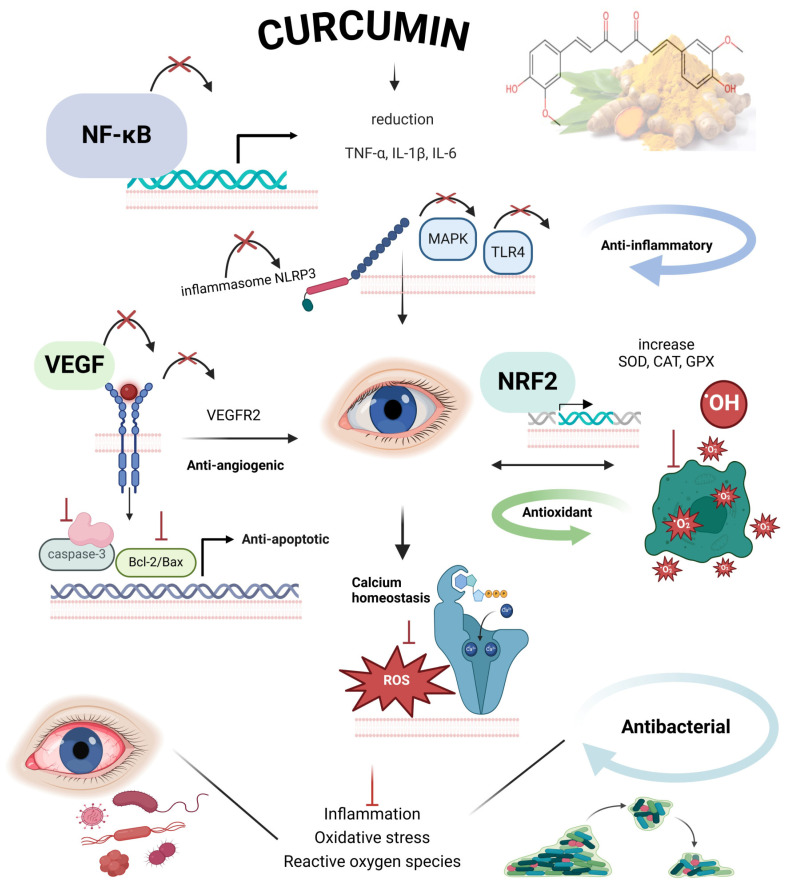
Potential mechanisms of action of curcumin. The diagram highlights key pathways, including NF-κB (nuclear factor kappa-light-chain-enhancer of activated B cells), Nrf2 (nuclear factor erythroid 2-related factor 2), NLRP3 (NOD-like receptor pyrin domain containing 3), MAPK (mitogen-activated protein kinase), TLR4 (toll-like receptor 4), and VEGF (vascular endothelial growth factor), and their roles in modulating inflammation, oxidative stress, angiogenesis, apoptosis, calcium homeostasis, and antibacterial activity.

Curcumin serves as a central modulator in multiple molecular systems involved in ocular health. It promotes a dynamic equilibrium between cellular processes that sustain ocular tissue integrity (Figure 2). This bioactive compound uniquely interacts with key signaling pathways, adjusting their intensity and function to reach an ideal homeostatic state.

For instance, its anti-inflammatory action arises from a strategic blockade of pro-inflammatory signals, such as nuclear factor kappa-light-chain-enhancer of activated B cells (NF-κB) and NLR family pyrin domain containing 3 (NLRP3), leading to a reduction in the excessive production of cytokines like tumor necrosis factor-alpha (TNF-α), interleukin 1 beta (IL-1β), and interleukin 6 (IL-6). This effect can be likened to the fine-tuning of an inflammatory thermostat, ensuring that inflammation required for tissue repair is preserved while preventing the collateral damage associated with chronic inflammation [38,48,49,50,51].

In the antioxidant domain, curcumin stands out by activating nuclear factor erythroid 2-related factor 2 (NRF2), a regulatory protein that coordinates the expression of antioxidant enzymes, including superoxide dismutase (SOD), catalase, and glutathione peroxidase (GPX). This mechanism not only neutralizes reactive oxygen species (ROS) but also preserves mitochondrial metabolic functions, protecting ocular cells from oxidative stress. Thus, curcumin acts as a metabolic sentinel, preventing cumulative damage over time [52,53,54,55,56,57,58].

Furthermore, its ability to inhibit vascular endothelial growth factor (VEGF) in angiogenic processes is particularly relevant in conditions characterized by pathological neovascularization, such as DR and age-related macular degeneration (AMD). This effect is not merely inhibitory but restorative, realigning angiogenic processes to meet the tissue’s physiological needs. Curcumin’s regulation of the apoptotic balance through modulators like B-cell lymphoma 2 (Bcl-2)/Bcl-2-associated X protein (Bax) ratio further solidifies its role as a cellular protector, preventing uncontrolled cell death while maintaining the selective elimination of damaged cells [52,55,56,59,60,61,62,63,64].

Curcumin also exerts a significant antibacterial effect through its modulation of ROS and its ability to suppress bacterial cell wall synthesis. By neutralizing ROS and interfering with bacterial biofilm formation, curcumin enhances its antibacterial potential. This mechanism reduces bacterial load while maintaining a controlled inflammatory response. Through the modulation of the immune system, curcumin minimizes excessive inflammatory damage, contributing to a more effective defense mechanism against microbial infections [38,48].

Finally, curcumin’s immunomodulatory effects ensure a favorable environment for tissue repair and regeneration, particularly in autoimmune and infectious diseases affecting the eye. This comprehensive action, combined with its extracellular matrix stabilization properties and regulation of endoplasmic reticulum stress, positions curcumin as a molecularly orchestrated intervention with therapeutic potential adaptable to a wide range of ocular conditions [24,49,50,51].

### 2.1. Retinal Diseases

Retinal diseases, characterized by complex pathological processes, such as inflammation, oxidative stress, and pathological angiogenesis, are major contributors to vision impairment. Leveraging curcumin’s unique ability to target these mechanisms, recent research highlights its potential in mitigating retinal damage and preserving visual function (Table 2) [2].

In DR, curcumin alleviates hyperglycemia-induced damage to retinal pigment epithelium (RPE) cells and helps maintain blood–retinal barrier integrity. A recent experimental study in diabetic rats showed that curcumin reduces pro-inflammatory cytokines (TNF-α, IL-1, and IFN-γ) and oxidative stress markers, such as malondialdehyde (MDA), GPX, CAT, and SOD [48,65]. These findings align with its broader molecular actions, including modulation of extracellular signal-regulated kinase (ERK) and Akt (protein kinase B, PKB) pathways to protect RPE cells and inhibit retinal neovascularization, inhibition of chronic inflammation via NF-κB suppression, and oxidative stress reduction [48,65,66].

In addition to DR, inflammation is a key driver in retinal diseases such as age-related macular degeneration (AMD) and best vitelliform macular dystrophy (BVMD). Curcumin suppresses pathways like NF-κB, reducing pro-inflammatory cytokines (TNF-α, IL-1β, and IL-6) and mitigating chronic inflammation [1,16]. This anti-inflammatory effect, mediated through NF-κB modulation, is a common mechanism also observed in glaucoma, where curcumin regulates the NF-κB pathway to reduce inflammation and oxidative stress, helping protect retinal ganglion cells (RGCs) and improve the optic nerve integrity [53,67].

Curcumin’s antioxidant effects are critical in combating oxidative stress, a major contributor to retinal degeneration. By enhancing endogenous antioxidant defenses, such as SOD and catalase, curcumin protects retinal cells from ROS, which are implicated in diseases like retinitis pigmentosa (RP), AMD, and BVMD. It also reduces oxidative stress and light-induced damage, especially relevant in BVMD [52,53,54,55]. In all these conditions, curcumin also acts in glaucoma, where its ability to reduce ROS levels protects RGCs and modulates antioxidant pathways, such as the activation of Nrf2, which is critical for protecting cells against ROS-induced damage [53,56].

Furthermore, curcumin may regulate calcium homeostasis in RPE cells, enhancing their function and survival, especially in BVMD [55]. Neuroprotective effects of curcumin also extend to glaucoma, where it reduces RGC death by inhibiting caspase-3 activation and modulating factors such as Bcl-2 and Bax, providing additional protection against neuronal degeneration [56]. Curcumin’s ability to modulate autophagy offers a dual benefit by not only preventing cellular degeneration but also enhancing the clearance of toxic protein aggregates and damaged organelles in RPE cells. This dual action reinforces its potential as a therapeutic agent for retinal diseases, highlighting its capacity to target multiple pathological mechanisms simultaneously, thereby preserving retinal structure and function [68].

**Table 2:** Curcumin: molecular mechanisms and activity potential in retinal diseases.

Relevant Diseases	Activity Potential	Key Actions and Targets	Refs.
DR	BRB integrity and mitochondrial protection	Modulates ERK and Akt pathways, preserves mitochondrial function in RPE cells.	[48,65,66]
AMD, DR, GL	Anti-inflammatory	Suppresses pro-inflammatory cytokines (TNF-α, IL-1β, and IL-6).	[1,16,53,67]
AMD, RP, BVMD, GL	Antioxidant	Activates Nrf2 pathway, enhances glutathione synthesis and heme oxygenase-1 expression.	[52,53,54,55,56]
BVMD	Calcium homeostasis	Regulates calcium signaling through SERCA in RPE cells.	[55]
BVMD, GL	Anti-inflammatory and neuroprotective	Suppresses inflammation and apoptosis in RPE cells; modulates cytokines and NF-κB.	[55,56]
AMD, DR, RP, GL	Neuroprotective and anti-apoptotic	Modulates apoptotic regulators (Bcl-2 and Bax); suppresses caspase-3 activation.	[55,59,60,61]
AMD	Autophagy activation	Restores autophagy flux, modulates LC3-II/LC3-I ratio, protects RPE cells from degeneration.	[68]
Wet AMD, PVR, RVO, GL	Anti-angiogenic	Inhibits VEGF receptor phosphorylation (e.g., VEGFR2), limits endothelial cell migration.	[17,62,63,64,69,70,71,72,73,74]
PVR, BVMD, GL	Fibrosis regulation	Inhibits EMT via suppression of Smad-dependent TGF-β1 signaling.	[74,75,76,77,78]
RB	Anti-tumor	Upregulates miR-99a, modulates JAK/STAT.	[79]

Abbreviations: Akt—protein kinase B, PKB; AMD—age-related macular degeneration; Bax—Bcl-2-associated X protein; BVMD—best vitelliform macular dystrophy; BRB—blood–retinal barrier; DR—diabetic retinopathy; ECM—extracellular matrix; ERK—extracellular signal-regulated kinase; IL—interleukin; LC3-II/LC3-I—microtubule-associated protein 1 light chain 3 (II/I ratio); JAK/STAT—Janus kinase/signal transducer and activator of transcription; miR—microRNA; NF-κB—nuclear factor kappa-light-chain-enhancer of activated B cells; PVR—proliferative vitreoretinopathy; RB—retinoblastoma; RP—retinitis pigmentosa; RPE—retinal pigment epithelium; ROS—reactive oxygen species; RVO—retinal vascular obstruction; SERCA—sarcoplasmic/endoplasmic reticulum calcium ATPase; SOD—superoxide dismutase; TGF-β1—transforming growth factor beta 1; TNF-α—tumor necrosis factor alpha; VEGF—vascular endothelial growth factor; VEGFR2—vascular endothelial growth factor receptor 2.

For wet AMD and other vascular-related conditions, PVR and retinal vascular obstruction (RVO), curcumin’s anti-angiogenic activity inhibits VEGF, preventing abnormal blood vessel formation and mitigating retinal damage [17,62,64,69,70]. VEGF is essential for new blood vessel formation and vascular permeability, playing a key role in retinal diseases like DR, RVO, and exudative AMD. It is produced by retinal endothelial and pigment epithelial cells and is considered a key target in anti-angiogenic therapies. For instance, VEGF-A, a key driver in wet AMD progression, binds to VEGFR2, promoting angiogenesis and vascular leakage [64,71,72]. Although glaucoma is not primarily an angiogenic disease, curcumin’s inhibition of VEGF may contribute to retinal vascular protection, especially in ischemic or injury contexts, with a positive impact on intraocular pressure regulation [63,73,74].

Curcumin also exhibits neuroprotective and anti-apoptotic effects, reducing ganglion cell death and modulating apoptotic pathways. These effects are particularly significant in DR, AMD, and BVMD, where retinal cell survival is crucial for preserving vision [55,59,60,61]. This mechanism is also present in glaucoma, where curcumin exerts similar effects to protect optic nerve cells from programmed cell death, an important feature of glaucoma pathogenesis [53,67].

Curcumin regulates fibrosis, a hallmark of PVR and potentially BVMD, by inhibiting TGF-β1 activity and suppressing miR-21, a microRNA that promotes fibrogenesis, thereby further inhibiting the fibrotic process [55,75,78]. This inhibition reduces the expression of fibrosis-related proteins, such as α-smooth muscle actin (α-SMA), type I collagen (COL1A1), and type III collagen (COL3A1), and potentially preserving retinal structure [55,75,76,77]. In glaucoma, curcumin has shown a similar effect by modulating fibrotic processes associated with optic nerve injury through the regulation of TGF-β1 and other fibrosis-related molecular factors [63,74].

Lastly, curcumin has demonstrated promising therapeutic potential in retinoblastoma (RB), the most common malignant intraocular tumor in children. It exerts anti-tumor effects by inhibiting cell proliferation, migration, and invasion while promoting apoptosis. These effects are primarily mediated through the upregulation of microRNA (miR-99a), which negatively regulates the Janus kinase/signal transducer and activator of transcription (JAK/STAT) signaling pathway, a crucial pathway involved in tumor progression and cell survival. This suggests that curcumin’s modulation of miRNA expression contributes significantly to its anti-cancer properties, making it a candidate for adjunct therapy in RB treatment [79].

### 2.2. Corneal Diseases

The cornea faces distinct challenges, including infections, fibrosis, and inflammation, which can compromise its transparency and refractive function. In this context, curcumin’s ability to modulate oxidative stress, inflammation, and angiogenesis has shown promise in addressing these conditions (Table 3).

Inflammatory processes are central to keratitis and DED. Curcumin effectively inhibits p38 MAPK and NF-κB signaling, reducing pro-inflammatory cytokines like IL-1β, IL-6, and TNF-α. A study showed curcumin (5 μM) completely abolished hyperosmoticity-induced IL-1β elevation in human corneal epithelial cells [48].

Oxidative stress exacerbates corneal injury and delays healing. Curcumin’s antioxidant capacity neutralizes ROS, protecting corneal cells and enhancing cellular survival under stress conditions. Guo et al. demonstrated that pretreatment with 12.5 µM curcumin enhances antioxidant defenses, including SOD1 and heme oxygenase-1, via the Keap1/Nrf2/ARE pathway, improving cell survival under oxidative stress [57].

Curcumin also promotes corneal healing by stimulating cell migration and collagen synthesis, which reduces scarring and preserves corneal transparency. A dose-dependent effect was observed, where curcumin (10.0–12.5 mg/L) inhibited keratocyte proliferation and modulated fibrotic markers, upregulating decorin and CD90 (a glycoprotein marker of activated fibroblasts associated with tissue remodeling) while downregulating keratocan and aldehyde dehydrogenase [80].

**Table 3:** Curcumin: molecular mechanisms and activity potential in corneal diseases.

Relevant Diseases	Activity Potential	Key Actions and Targets	Ref.
Keratitis, dry eye disease	Anti-inflammatory	Inhibits p38 MAPK and NF-κB signaling, reducing cytokine production.	[48]
Keratitis, dry eye disease	Oxidative stress reduction	Reduces ROS, protecting epithelial and endothelial cells.	[57]
Corneal fibrosis, keratitis	Promotes healing andReducing fibrosis	Enhances cell migration and collagen synthesis, reducing scarring.	[80]
Corneal fibrosis	Anti-angiogenic	Inhibits corneal neovascularization by suppressing VEGF.	[81]

Abbreviations: MAPK—mitogen-activated protein kinase; NF-κB—nuclear factor kappa-light-chain-enhancer of activated B cells; ROS—reactive oxygen species; VEGF—vascular endothelial growth factor.

In corneal neovascularization, a severe complication often associated with corneal diseases, curcumin’s anti-angiogenic properties offer significant therapeutic advantages. By downregulating VEGF, curcumin impedes the formation of abnormal blood vessels, thereby reducing tissue damage and preserving vision. In an alkaline-burned rat model, the topical application of 40 μmol/L curcumin every 12 h for five days significantly reduced the area of new blood vessels compared to controls, showcasing its potential for managing angiogenesis-related corneal pathologies [81].

### 2.3. Bacterial Ocular Diseases

Bacterial ocular infections, such as conjunctivitis, keratitis, and endophthalmitis, often involve severe inflammatory responses, biofilm formation, and resistance to conventional antibiotics. Curcumin’s molecular mechanisms address these challenges by targeting inflammation, oxidative stress, and bacterial survival strategies (Table 4).

Biofilm formation is a critical factor in bacterial persistence and resistance. Curcumin disrupts biofilm matrix integrity and inhibits bacterial efflux pumps, thereby increasing susceptibility to antibiotics. This effect is particularly relevant against multidrug-resistant (MDR) pathogens, including MRSA and *Pseudomonas aeruginosa* [15].

Inflammation, a hallmark of bacterial ocular diseases, is modulated by curcumin through the inhibition of NF-κB and MAPK pathways, reducing cytokine storms and promoting ocular tissue recovery. In conjunctivitis, curcumin-based formulations like the product Haridra^®^ have shown anti-inflammatory and antibacterial efficacy [82].

Oxidative stress exacerbates tissue damage during bacterial infections. Curcumin’s ability to neutralize ROS through the activation of the Keap1/Nrf2/ARE pathway preserves epithelial and retinal cell viability under stress conditions [58].

Additionally, emerging research on the gut-ocular axis has opened new avenues for understanding how systemic factors, such as the gut microbiota, influence ocular immunity. Dietary interventions, including omega-3 fatty acids, carotenoids, and probiotics, have been shown to modulate both systemic and ocular immunity, reducing inflammation and improving overall eye health. Curcumin, with its anti-inflammatory and antioxidant effects, integrates into these strategies, offering a multifaceted approach to managing bacterial ocular diseases [24].

**Table 4:** Curcumin: molecular mechanisms and activity potential in bacterial diseases.

Relevant Diseases	Activity Potential	Key Actions and Targets	Ref
Endophthalmitis	Anti-inflammatory	Reduces cytokine storms, protects retinal cells by modulating MAPK and NF-κB pathways.	[37]
Conjunctivitis	Anti-inflammatory, antibacterial	Inhibits NF-κB signaling, reduces IL-1β and TNF-α, disrupts bacterial membranes.	[82]
Keratitis	Antioxidant, antibacterial	Neutralizes ROS, inhibits MMP-9, disrupts biofilm matrix.	[58]

Abbreviations: MAPK—mitogen-activated protein kinase; MMP-9—matrix metalloproteinase 9; MRSA—methicillin-resistant *Staphylococcus aureus*; NF-κB—nuclear factor kappa-light-chain-enhancer of activated B cells; ROS—reactive oxygen species.

### 2.4. Periocular and Ocular Surface Disorders

Chronic inflammation, immune dysregulation, and oxidative stress characterize eyelid diseases, such as blepharitis, blepharospasm, and eyelid dermatitis. Curcumin addresses these through its multi-targeted mechanisms, offering significant therapeutic potential (Table 5).

Curcumin suppresses NF-κB and TLR4 signaling pathways, reducing pro-inflammatory cytokines like TNF-α and IL-1β. Additionally, it inhibits inflammasome activity, specifically NRLP3 to reduce tissue damage in blepharitis and dermatitis. Its antioxidant activity neutralizes ROS, mitigating oxidative tissue damage, while modulation of Th17 cell activity reduces immune dysregulation in autoimmune eyelid conditions [49,50,51].

**Table 5:** Curcumin: molecular mechanisms potential in periocular and ocular surface disorders.

Relevant Diseases	Activity Potential	Key Actions and Targets	Refs.
Blepharitis, eyelid dermatitis	NF-κB Suppression	Reduces pro-inflammatory cytokines (TNF-α, IL-1β, and IL-6); Suppresses NRLP3 activity, reducing tissue damage.	[49,50,51]
Autoimmune eyelid disorders	Immune Modulation	Decreases Th17 activity and dendritic cell response; TLR4, MAPK, NF-κB.	[50]
Chronic inflammation, dermatitis	Antioxidant Defense	Neutralizes free radicals, enhancing tissue resilience; glutathione, SOD, and catalase.	[83]
Blepharospasm	Neuroprotective Effects	Modulates neuroinflammation and oxidative stress.	[83]
AllergicConjunctivitis	Reduction of Inflammatory Markers	Suppresses key mediators, such as IL-4, IL-5, and iNOS, in ocular tissues.	[84]
Meibomian gland Dysfunction	Anti-inflammatory, Lipid Regulation	Reduces inflammation in meibomian glands; modulates lipid composition in the tear film.	[85,86]
Dry Eye Disease	Oxidative Stress Modulation, Mucin Enhancement	Reduces oxidative stress, stabilizes tear film, enhances mucin production, and improves ocular comfort.	[85,86]

Abbreviations: MAPK—mitogen-activated protein kinase; NF-κB—nuclear factor kappa-light-chain-enhancer of activated B cells; NRLP3—NOD-like receptor family pyrin domain containing 3; SOD—superoxide dismutase; TLR4—toll-like receptor 4; VEGF—vascular endothelial growth factor.

In blepharospasm, curcumin exhibits neuroprotective effects by reducing neuroinflammation and oxidative stress, promoting cellular resilience [49,51,83]. Additionally, curcumin demonstrates potential in managing allergic conjunctivitis, an inflammatory condition of the ocular surface driven by Th2 immune responses. Studies indicate that curcumin reduces IgE-mediated inflammation, suppressing eosinophilic infiltration and Th2 cytokine production, such as IL-4 and IL-5, in the conjunctiva [84]. These actions extend curcumin’s therapeutic scope to inflammatory and immune-related ocular surface disorders.

Further expanding its utility, curcumin shows promise in treating meibomian gland dysfunction (MGD), a leading cause of ocular discomfort, by reducing inflammation and improving the lipid composition of the tear film. In dry eye disease, which is often linked with MGD, curcumin alleviates inflammation and oxidative stress while enhancing mucin production, stabilizing the tear film, and improving ocular comfort [85,86].

These combined actions position curcumin as a promising agent for managing complex periocular and ocular surface disorders.

## 3. Comprehensive Drug Delivery Systems for Curcumin in Ophthalmology

**Figure 3:**
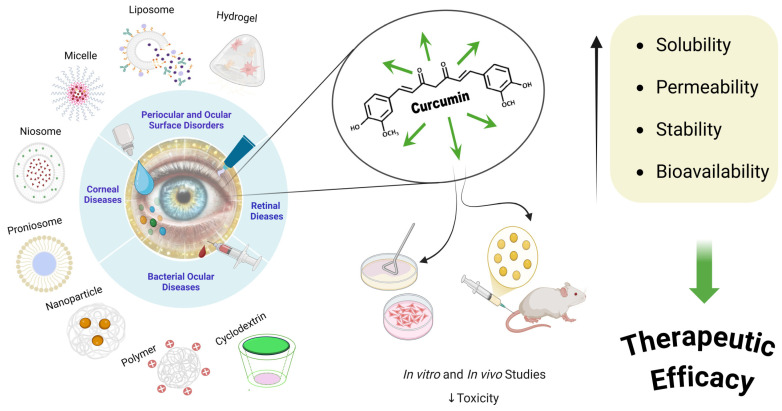
Schematic representation of curcumin-based ophthalmic drug applications: advanced delivery systems for ocular diseases.

Curcumin predominantly exists in the keto-enol form in polar solvents and is hydrophobic, being insoluble in water but soluble in organic solvents. This characteristic poses challenges for its therapeutic application. However, advanced drug delivery systems, such as encapsulation, have significantly improved curcumin’s solubility and stability in aqueous environments (Figure 3).

### 3.1. Non-Ionic Surfactant-Based Delivery Systems

Non-ionic surfactants enhance the bioavailability of hydrophobic drugs, especially curcumin, by forming stable and biocompatible carriers. These systems improve curcumin’s solubility, stability, and ocular residence time, thereby reducing systemic toxicity and enhancing therapeutic outcomes (Table 6).

Composed of non-ionic surfactants and cholesterol, niosomes enhance curcumin’s solubility, stability, and ocular residence time, reducing systemic toxicity [87]. Nanoemulsion-based formulations improve drug dispersion in aqueous environments, enhancing curcumin’s corneal permeability and bioavailability. This approach ensures rapid therapeutic action, crucial for treating acute ocular conditions [88]. Liposomes, a subclass of non-ionic surfactant carriers, enhance curcumin’s bioavailability and ocular distribution, supporting its use in retinal degeneration and inflammation [11,89].

In addition, curcumin-loaded proniosomal gels have emerged as a promising alternative to traditional corticosteroid treatments for ocular inflammation. These formulations, composed of surfactants, such as cremophore RH, lecithin, and cholesterol, offer high encapsulation efficiency (96%) and 3.22-fold greater permeability than conventional dispersions. Comparative in vivo studies demonstrated that these proniosomal gels significantly reduced inflammatory symptoms, achieving complete recovery within four days, comparable to corticosteroid drops. Curcumin’s natural origin minimizes adverse effects, such as intraocular pressure elevation, positioning it as a safer yet equally effective anti-inflammatory treatment option. Curcumin encapsulated in proniosomal gel demonstrated exceptional biocompatibility and safety, coupled with potent anti-inflammatory properties. This innovative formulation enhanced ocular retention and significantly improved corneal permeability, delivering a sustained release effect over 24 h [30].

Recent research underscores the value of transferosomes (TFSs), ultra-deformable vesicles comprising lipid bilayers and surfactants, such as Tween 80. TFS exhibits exceptional drug entrapment efficiency (>99%) and enhances drug penetration across corneal and conjunctival barriers, enabling deeper and more effective delivery to ocular tissues. They also improve precorneal retention, ensuring sustained therapeutic levels and superior bioavailability. Studies highlight their excellent compatibility with ocular tissues and their potential to optimize the delivery of curcumin and similar compounds in treating eye diseases [90].

**Table 6:** Curcumin: non-ionic surfactant-based delivery systems.

Type of Carriers	Activity Potential	Key Actions and Targets	Advancements	Refs.
Liposomes	Controlled release enhanced ocular bioavailability.	Improved ocular distribution, sustained release.	Stability in aqueous solutions.	[11,89]
Nanoemulsions	Increased solubility and bioavailability.	Rapid and efficient release.	Excipient stability and safety considerations.	[88]
Niosomes	Enhanced encapsulation efficiency, reduced toxicity.	High permeability and stability in the ocular tissues.	Alternative to corticosteroids with improved safety.	[30,90]

Additionally, lipophilic vehicles, such as medium-chain triglycerides (MCTs) and squalane, demonstrate varying degrees of effectiveness. Ex vivo studies reveal that squalane suspensions notably enhance curcumin’s penetration into ocular tissues compared to MCT solutions, emphasizing the role of vehicle partitioning in optimizing drug delivery [91].

Polymeric in situ gelling systems represent an innovative approach for ocular curcumin delivery. These inserts, composed of biocompatible polymers such as HPMC, CMC, and Pluronic F127, provide sustained drug release and enhanced mucoadhesion. Characterization studies show that curcumin in these systems is dispersed molecularly, with smooth and uniform surfaces. Importantly, the inserts exhibit superior corneal permeation (5.4- to 8.86-fold increase) and retention times compared to conventional suspensions. These properties underscore their potential to replace traditional eye drops, offering improved therapeutic efficacy through prolonged action and reduced dosing frequency [92].

### 3.2. Nanoparticle-Based Systems

Nanoparticles provide controlled drug release and enhanced stability for curcumin, making them suitable for various ocular conditions (Table 7).

Solid lipid nanoparticles ensure high drug loading, stability, and minimal systemic toxicity. They have demonstrated effectiveness in reducing oxidative stress in retinal cells, addressing posterior segment eye diseases, such as AMD and DR [93].

Polymeric nanoparticles, such as PLGA(polylactic-co-glycolic acid)-based nanoparticles, enhance curcumin’s stability and bioavailability. An innovative approach involves biodegradable scleral plugs, which enable sustained drug release for up to 14 days. Studies show that scleral plugs with curcumin concentrations of 0.5 mg, 1.0 mg, and 1.5 mg achieve therapeutic levels (above 15 µg/mL) in vitro, with no adverse effects observed in vivo models, such as changes in intraocular pressure or retinal integrity. These findings underscore the safety and efficacy of scleral plugs for posterior ocular diseases [94].

A promising strategy to address curcumin’s limitations involves using diphosphorylated curcumin (Cur-2p), a prodrug that generates curcumin nanoparticles in situ. This approach enhances curcumin’s stability and reduces aggregation in water. Upon enzymatic conversion by alkaline phosphatase (ALP) in cancer cells, Cur-2p exhibits selective cytotoxicity against ALP-overexpressing cancer cells while sparing normal cells. Additionally, intravitreal injections of Cur-2p demonstrate superior intraocular biocompatibility, preserving retinal morphology and function. In a rodent model of uveitis, Cur-2p effectively suppresses inflammation, outperforming unmodified curcumin. These findings highlight Cur-2p’s potential as a next-generation nanoparticle-based system for ocular drug delivery [95].

Mixed micelle in situ gels, composed of Pluronic P123 and D-α-tocopherol polyethylene glycol succinate, have been developed to overcome curcumin’s poor water solubility and limited corneal permeability. The optimized micellar formulations, when combined with gellan gum, form transparent in situ gels that sustain drug release while enhancing corneal retention. In vitro studies confirmed a sustained drug release profile, while ex vivo corneal permeation tests demonstrated superior drug delivery, with cumulative drug permeation up to 1.32 times higher compared to standard curcumin solutions [10]. Similarly, curcumin-loaded mixed micelle in situ gel (Cur-MM-ISG) improves ocular drug delivery. The system combines small, stable micelles with gellan gum to form a transparent gel upon application. Compared to free curcumin, Cur-MM-ISG significantly enhanced corneal permeation and retention time without causing irritation, highlighting its potential for sustained and efficient ocular therapy. This system supports sustained therapeutic effects with excellent ocular tolerance [39].

Thermosensitive gels (CUR-CNLC-GEL) formulation demonstrated promising results in improving the bioavailability of curcumin for ocular therapy. With a sol-gel transition temperature of 34 °C, it ensured practical application. The nanogel exhibited 1.56-fold higher permeability and a 9.24-fold increase in bioavailability (AUC_0→∞_) compared to a conventional curcumin solution. Additionally, the enhanced C_max_ and prolonged mean residence time (MRT) indicated effective controlled release and retention properties. These attributes the position CUR-CNLC-GEL as a highly promising candidate for next-generation ocular drug delivery, offering superior corneal permeation and extended therapeutic efficacy [96].

Thermosensitive hydrogels, especially when paired with nanoparticles, offer an innovative approach to achieving sustained drug release. For instance, hydrogels incorporating curcumin-loaded nanoparticles (CUR-NPs) and latanoprost have demonstrated notable improvements in bioavailability for glaucoma therapy. This dual-drug delivery system addresses oxidative stress in trabecular meshwork cells, effectively mitigating inflammation, reducing mitochondrial ROS production, and decreasing apoptosis levels. Additionally, it enhances both uveoscleral and trabecular outflow, highlighting its potential as a promising treatment for glaucoma. This system enables the development of topical eye drops capable of sustaining drug release for up to 7 days, enhancing residence time in the rabbit eye, and improving corneal permeation with minimal toxicity [97].

The study on the development of a thermoresponsive ophthalmic in situ gel containing curcumin-loaded albumin nanoparticles (Cur-BSA-NPs-Gel) presents significant advancements in ocular drug delivery systems. By optimizing the formulation through a central composite design, the researchers achieved a gel that transitions from liquid to semi-solid under physiological conditions, ensuring easy application and sustained drug release. Incorporating albumin nanoparticles minimally impacted the gel’s structure while enhancing curcumin’s bioavailability in the aqueous humor, as confirmed by in vivo studies in rabbit models. The formulation demonstrated safety for ophthalmic use, with no signs of eye irritation, and offers potential for prolonged therapeutic effects, making it a promising candidate for ocular treatments [98].

Recent advancements have introduced dissolvable hybrid microneedles (MNs) patches as a novel method for ocular delivery of curcumin. These patches incorporate curcumin-loaded polymeric micelles into a hyaluronic acid matrix, using a micromolding process to ensure efficient drug dispersion. Studies reveal that this system facilitates sustained drug release over eight hours and extends pre-corneal retention to more than 3.5 h, significantly improving bioavailability. MNs patch can create temporary microchannels in the corneal epithelium, enhancing permeability. In vivo testing demonstrated its superior efficacy in treating endotoxin-induced uveitis, reducing inflammatory cell infiltration more effectively than conventional eye drops, making it a promising tool for managing intraocular inflammatory disorders [99].

The development of curcumin-loaded nanostructured lipid carriers (CUR-NLC) coated with thiolated chitosan (CS-NAC) offers a promising solution for topical ocular drug delivery. This innovative system achieves sustained drug release for up to 72 h and significantly enhances corneal permeability and retention. Compared to coatings using chitosan oligosaccharides (COS) and carboxymethyl chitosan (CMCS), the CS-NAC coating demonstrated superior performance, with permeability coefficients increasing by 6.4 and 18.8 times relative to uncoated CUR-NLC and conventional eye drops, respectively. Furthermore, ocular irritation tests confirmed the biocompatibility of CS-NAC-CUR-NLC [40].

**Table 7:** Curcumin: nanoparticle-based delivery systems.

Type of Carriers	Activity Potential	Key Actions and Targets	Advancements	Ref
Lipid Nanoparticles	Controlled release, high stability.	Reduced oxidative stress in retinal cells.	Large-scale production feasibility.	[93]
Polymeric Nanoparticles	Biodegradable material, precise release control.	Effective in retinal diseases, such as AMD.	Evaluated for safety and prolonged therapeutic efficacy.	[94]
Nanomicelles	Improved solubility and antioxidant properties.	Robust ocular permeability, reduced inflammation.	Promising in ocular inflammation management.	[100]

Abbreviations: AMD—age-related macular degeneration.

The formulation of curcumin-loaded nanostructured lipid carriers (NLCs) using hot-melt emulsification and ultrasonication has demonstrated significant potential for ocular drug delivery. Optimized through a central composite design, the resulting NLCs showcased a particle size of approximately 66.8 nm, high encapsulation efficiency (96%), and consistent drug loading. These properties contributed to enhanced stability over three months at low temperatures and superior transcorneal permeability. Ex vivo tests revealed a 2.5-fold increase in curcumin permeation across rabbit corneas compared to standard formulations, without evidence of adverse effects, underscoring the NLCs’ ability to improve drug delivery efficiency while maintaining safety [101].

The development of a nanomicelle-based curcumin formulation utilizing a PVCL-PVA-PEG graft copolymer has shown promise for enhancing ocular drug delivery. This system significantly improves curcumin’s solubility and stability while offering robust antioxidant activity. In vitro and in vivo studies demonstrated that these nanomicelles enhance cellular uptake and corneal permeation compared to free curcumin solutions. Additionally, the formulation exhibited excellent ocular tolerance, with no signs of irritation in rabbit models. These results suggest that nanomicelles could serve as an effective platform for delivering curcumin topically in the treatment of ocular inflammation and related conditions [100].

Recent advancements in curcumin-based therapies for AMD highlight the promise of aqueous nanomicellar formulations (CUR-NMF). This innovative approach overcomes curcumin’s poor aqueous solubility, a key limitation for its therapeutic use. CUR-NMF, developed using hydrogenated castor oil (HCO-40) and octoxynol-40 (OC-40), offers a stable delivery system optimized for retinal protection. Studies demonstrate its antioxidant, anti-inflammatory, and anti-angiogenic effects, showing significant protection against oxidative stress in retinal cells and a reduction in VEGF release, a critical factor in AMD pathology. Furthermore, sustained drug release profiles and favorable safety assessments suggest CUR-NMF could provide long-term therapeutic benefits for both dry and wet AMD [102].

### 3.3. Cyclodextrin Complexes

**Figure 4:**
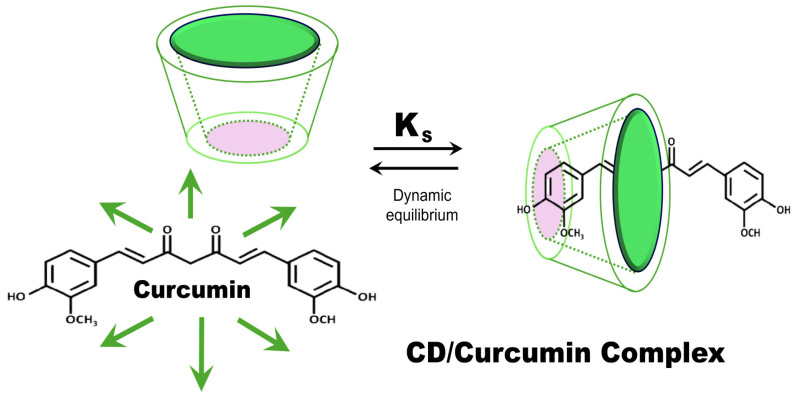
Schematic representation of inclusion complexes involving curcumin and CDs.

Cyclodextrins (CDs) play a crucial role in enhancing curcumin’s solubility, stability, and bioavailability through the formation of inclusion complexes (Figure 4, Table 8). These complexes significantly improve curcumin’s therapeutic effectiveness in both anterior and posterior ocular diseases. Among the CDs, β-cyclodextrins (βCD) and γ-cyclodextrins (γCD) stand out due to their higher solubility, capacity to self-assemble into nanoaggregates, and favorable safety profile for ocular applications [103,104].

Since the mid-1990s, CD-based inclusion complexes have provided significant technological advantages for pharmaceutical formulations. By enhancing the stability, solubility, and bioavailability of bioactive compounds, these complexes address key challenges in drug delivery. A notable example is the use of cyclodextrins to improve the solubility and stability of chloramphenicol, a patented formulation still in use today [105]. This approach enabled the development of stable pharmaceutical solutions and optimized separation processes, underscoring the enduring relevance of this technology [106].

Methyl-β-cyclodextrin (M-β-CD) is the cyclodextrin used in commercial eye drop formulations, including those containing chloramphenicol. Hydroxypropyl-β-cyclodextrin (HP-β-CD) is also covered by this patent for chloramphenicol due to its superior solubility and biocompatibility [105]. More recently, CD-based inclusion complexes have been explored for cannabinoid delivery, demonstrating promising potential in pain management and anti-inflammatory therapies. These developments highlight the versatility of cyclodextrins in modern drug delivery, particularly for ocular applications, where solubility, stability, and bioavailability are critical for therapeutic success [107].

Modified CDs, such as ethylene diamine (EDA)-modified βCD, have demonstrated superior capabilities in improving curcumin (CUR) solubility and stability. These complexes provide enhanced thermodynamic properties, making CUR more bioavailable for ocular applications. Curcumin-EDA-βCD nanoparticles exhibit excellent corneal permeability, as shown in vitro porcine cornea experiments, and maintain high biocompatibility, confirmed by histological analyses of porcine corneas and bovine corneal epithelial cell viability. These properties make them particularly suitable for addressing anterior segment diseases like keratitis and dry eye disease [104].

In a recent study, various CD-curcumin complexes were prepared and characterized, showing significant improvements in solubility. The freeze-drying method produced highly soluble complexes, and the optimal formulation provided sustained release for over 96 h. This approach offers a promising solution for curcumin’s use in ocular therapies, such as eye drops for conditions like retinitis pigmentosa [108].

Inclusion complexes of CUR with hydroxypropyl-β-cyclodextrin (HP-βCD) were successfully developed using the cosolvency/lyophilization method, resulting in significant improvements in CUR solubility, stability, and therapeutic efficacy. The complexes demonstrated superior antioxidant and anti-inflammatory activities compared to free CUR. To facilitate ocular administration, an in-situ gel system was prepared using Pluronic F127 and chitosan, providing mucoadhesion and sol-gel transition between 26–35 °C. Viscosity, pH, and clarity tests confirmed the system’s suitability for ocular application. In vitro release studies showed sustained drug release for 6 h, fitting the Weibull kinetic model. This approach offers a promising drug delivery strategy for ocular diseases, supporting prolonged and controlled drug release [109].

The poor solubility and stability of CUR limit its application in ocular drug delivery. To address this, CUR was complexed with βCD and HP-βCD using co-solvent, sonication, and freeze-drying methods in 1:1 and 1:2 molar ratios. The freeze-drying method produced the most water-soluble complexes. Among the 12 tested formulations, the F11 formulation, prepared with pH 6.8 phosphate buffer containing 1% Tween 80, demonstrated sustained drug release for over 96 h. The drug release followed a Higuchi non-Fickian diffusion model. These findings suggest that F11 could be developed as a once-daily eye drop formulation, offering a promising approach for the sustained delivery of curcumin in the treatment of ocular diseases, such as retinitis pigmentosa [110].

The use of CDs to optimize corneal penetration of CUR has shown promising results. In ex vivo models using porcine corneas, the combination of CDs with nanoparticles demonstrated greater drug permeation. This improvement is attributed to the ability of CDs to form inclusion complexes, enhancing curcumin’s solubility and stability, while nanoparticles enable sustained release and protection against enzymatic degradation [111].

Recent studies have demonstrated that curcumin-loaded hydrogels, such as those incorporating CUR nanoparticles encapsulated with βCD and hyaluronic acid, accelerate corneal healing in ulcerative keratitis. This system not only improves corneal clarity and reduces inflammation but also enhances the quality of healed tissues, requiring fewer applications compared to conventional treatments. These formulations hold promises for future therapeutic use in treating ulcerative keratitis and other ocular conditions, providing an innovative, herbal-based alternative to traditional treatments [112].

The penetration of CUR into the cornea was evaluated using an ex vivo porcine eye model and a digital image analysis technique. Several formulation strategies, including oily solutions, oily suspensions, micelles, liposomes, nanosuspensions, and CD complexes, were explored to improve CUR corneal permeability. The results revealed that cyclodextrin-based formulations exhibited superior corneal penetration compared to other delivery systems. The image analysis approach effectively measured CUR penetration into corneal tissues, supporting the potential of cyclodextrin complexes as a delivery strategy for hydrophobic drugs in ocular applications. This technique offers a novel approach for optimizing the penetration of CUR and similar compounds into the cornea [111].

Among the most effective formulations are those based on modified βCDs and conjugates with tetrahydrocurcumin nanoparticles, which exhibited deeper penetration into ocular tissues. These strategies hold significant potential for treating both anterior and posterior segment ocular diseases, as they increase bioavailability and extend therapeutic effects [2,110].

γCD-based nanoparticles not only enhance drug permeation but also increase retention time on the ocular surface, promoting sustained drug release and reducing the frequency of administration. Additionally, the presence of tear enzymes like α-amylase facilitates drug release from γCD complexes, further boosting bioavailability. For instance, γCD-based eye drops containing dexamethasone achieved higher concentrations in ocular tissues compared to commercial formulations. Moreover, these formulations were well tolerated, with no significant ocular irritation or toxicity observed. γCD has also been employed in formulations for dorzolamide, telmisartan, and nepafenac, demonstrating improved pharmacokinetics and sustained drug release for up to 24 h [103].

Inclusion complexes of CUR with HP-βCD enhance curcumin’s solubility, dissolution rate, and bioavailability, essential for ocular drug delivery. Studies using co-evaporation methods revealed a 1:1 molar ratio complex with a solubility constant of 30.09 mM^−1^. Characterization techniques, such as XRD, confirmed the loss of curcumin’s crystalline structure, while FTIR and DTA indicated no chemical interactions. In vitro dissolution tests showed faster release of CUR from the complex compared to its pure form and physical mixtures. This approach improves curcumin’s bioavailability, making it a promising strategy for ocular drug delivery systems [113].

The ocular delivery of CUR faces significant barriers due to anatomical and physiological constraints; however, advances in nanoengineered systems have shown promising results. The formation of inclusion complexes with HP-CDs through spray-drying significantly enhanced the solubility, permeability, and stability of CUR. Enhanced corneal and retinal permeability was observed, along with increased antioxidant activity in ocular epithelial cells, including upregulation of SOD1, CAT1, and HMOX1. Moreover, protection against oxidative stress was confirmed in rabbit corneal tissues. These findings highlight the potential of CUR:HP-CD complexes to improve ocular drug bioavailability, thereby enhancing therapeutic outcomes for ocular diseases [110].

Cyclodextrin-based systems significantly enhance curcumin’s bioavailability, solubility, and therapeutic potential for ocular drug delivery. Advances in βCD, γCD, and HP-βCD systems, combined with nanoparticles or in situ gels, have demonstrated improved drug permeation, sustained release, and higher bioactivity. These strategies support the development of more effective ophthalmic treatments.

**Table 8:** Curcumin: cyclodextrin complex-based delivery systems.

Type of Carriers	Activity Potential	Key Actions and Targets	Advancements	Refs.
Β-Cyclodextrin Derivatives	Enhanced solubility and stability.	Improved corneal permeability and compatibility.	Address anterior segment diseases, such as keratitis.	[104]
Cyclodextrin-Nanoparticles	Targeted delivery, extended release.	Combats oxidative stress effectively.	Promising in posterior segment diseases management.	[2,110]
HP-βCD Inclusion Complex	Anti-inflammatory and antioxidant	Controlled release, mucoadhesion	In situ gel with sustained release for 6 h.	[109]
γCD-Nanoparticles	Sustained release and bioavailability	Tear enzyme-triggered release	Increased ocular retention, reduced dosage frequency.	[103]

### 3.4. Drug Delivery for Antibacterial Agents

Nanocomposites, such as cupriferous hollow nanoshells, combine silver and copper ions. These materials exhibit dual functionality: silver ions provide potent antibacterial activity, while copper ions promote tissue regeneration by stimulating fibroblast migration and angiogenesis. This dual approach is particularly beneficial in treating conditions like keratitis, where infections can impair spontaneous recovery and cause corneal damage. Nanocomposite-based treatments not only target the bacteria but also support the healing of damaged tissues, offering a comprehensive approach to managing complex infections [58].

Moreover, curcumin enhances traditional antibiotics by inhibiting bacterial efflux pumps and disrupting biofilms—critical mechanisms in antibiotic resistance. When combined with biopolymers like chitosan, curcumin has shown enhanced antibacterial effects, even at low concentrations, especially against resistant bacterial strains. This makes curcumin-based formulations a valuable tool in combating antibiotic-resistant ocular infections, such as conjunctivitis and keratitis [15,58,114].

Curcumin-based formulations have also demonstrated significant efficacy in the treatment of conjunctivitis. Products like Haridra^®^ and Ophthacare^®^ have been shown to combat pathogens like *Escherichia coli*, *Staphylococcus aureus*, *Klebsiella pneumoniae*, and *Pseudomonas aeruginosa* while also reducing inflammation and irritation. These formulations address not only the infection but also the underlying inflammation, providing a comprehensive treatment approach. Ophthacare^®^, which combines *Curcuma longa* with other herbal extracts, offers an effective solution for a range of ocular conditions, including dry eye and inflammatory conjunctival disorders [82].

Endophthalmitis, an intraocular infection characterized by extensive inflammation and retinal damage, has benefited from nanotechnology-based drug delivery systems. Hybrid frameworks that incorporate silver nanoparticles and photosensitizers have been developed to disrupt biofilms while preserving host tissues. These systems, combined with curcumin’s anti-inflammatory properties, can modulate cytokine storms, support retinal cell survival, and preserve ocular structures. This combination not only targets the infection but also helps to protect the delicate retinal tissues, improving patient outcomes [37,115].

Recent innovations in drug delivery systems have further amplified the therapeutic potential of curcumin. Nanoparticles and liposomes are particularly effective at enhancing curcumin’s bioavailability and ocular penetration, ensuring sustained therapeutic effects. For example, dual-drug nanofibers, which combine curcumin with antibiotics, have shown enhanced bactericidal activity and accelerated tissue regeneration in preclinical models of ocular infections. These advanced delivery systems ensure that curcumin reaches the target site effectively, offering continuous antimicrobial action and supporting tissue healing [116].

**Figure 5:**
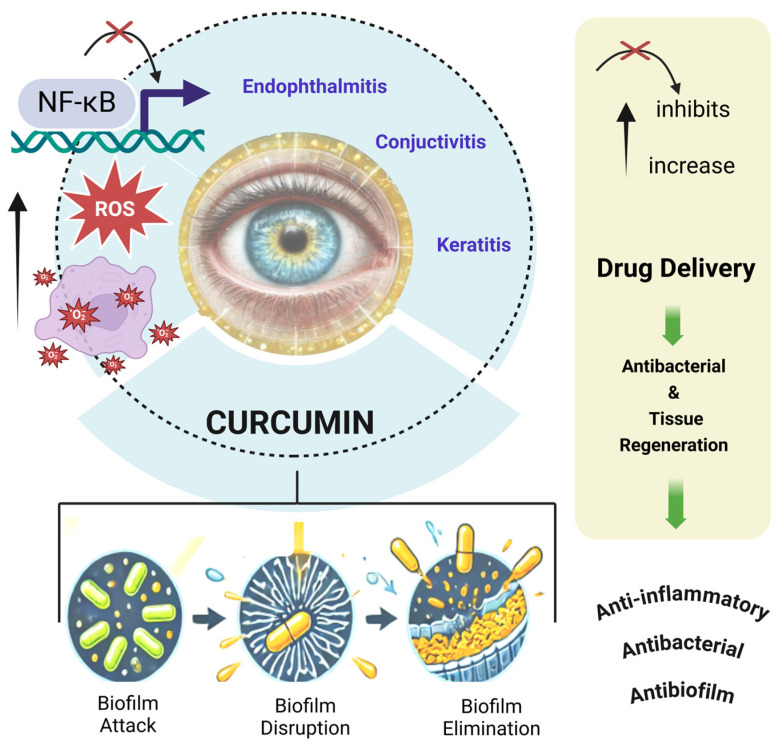
Curcumin: antibacterial and antibiofilm properties associated with drug delivery systems for the treatment of infectious diseases.

These systems not only enhance the effectiveness of conventional antibiotics but also provide innovative solutions to overcome the challenges posed by MDR bacteria, biofilms, and tissue damage (Figure 5). The integration of curcumin in these systems adds a further layer of therapeutic benefit, making it a promising tool in the management of ocular infections.

## 4. Emerging Research and Novel Approaches for Eyelid Conditions

### 4.1. Photodynamic Therapy (PDT)

Curcumin’s photosensitizing properties make it a promising candidate for PDT, targeting pathological cells in conditions such as ocular tumors and infections. This approach is particularly relevant for eyelid-specific conditions, offering [117,118,119]:

- Mechanisms: dual role as a photosensitizer and therapeutic agent.
- Applications: minimally invasive treatment for tumors, infections, and inflammatory disorders.
- Benefits: combines antioxidant and anti-inflammatory effects to enhance therapeutic outcomes for eyelid diseases.

### 4.2. Mucoadhesive Formulations

Mucoadhesive drug delivery systems, including hydrogels and films, prolong curcumin’s contact time with the ocular surface, increasing its therapeutic efficacy. These systems can be tailored to eyelid disorders such as the following [120,121,122]:

- Blepharitis and Dermatitis: prolonged retention enhances localized anti-inflammatory and antioxidant effects.
- Sustained Drug Release: mucoadhesive properties ensure better therapeutic outcomes for chronic eyelid conditions.
- Clinical Potential: effective for diseases requiring extended drug action, like anterior uveitis and diabetic retinopathy.

### 4.3. Neuroprotective Effects in Neurological Eyelid Disorders

Curcumin offers neuroprotective benefits by reducing neuroinflammation and promoting cell survival, with applications in [89,123]:

- Blepharospasm: reduces oxidative stress and neuroinflammation, alleviating involuntary twitching.
- Neuropathic Inflammation: modulates immune signaling, potentially relieving neuropathic eyelid pain.
- Mechanisms: targets inflammatory pathways (e.g., NF-κB and TLR4) and enhances antioxidant activity to protect against tissue damage.

## 5. Future Perspectives and Research Directions

Curcumin’s potential in eyelid diseases is supported by its potent anti-inflammatory, immunomodulatory, antioxidant, and antibacterial properties. The antibacterial activity of curcumin could be particularly beneficial in treating eyelid infections, such as those caused by Staphylococcus aureus or other bacterial pathogens, which are common in conditions like blepharitis and eyelid dermatitis. Future research should focus on developing targeted delivery systems, such as mucoadhesive and nanoparticle formulations, to enhance efficacy in localized eyelid treatments. Well-designed clinical trials are needed to validate curcumin’s safety and effectiveness in eyelid conditions, including blepharitis, blepharospasm, and eyelid dermatitis. Additionally, exploring combination therapies that integrate curcumin with conventional treatments or other phytochemicals could provide solutions for refractory eyelid conditions.

Given curcumin’s versatility as a therapeutic agent and advances in drug delivery technologies, it holds significant promise for addressing unmet needs in eyelid disease treatment.

## 6. Conclusions

Curcumin demonstrates significant therapeutic potential in ophthalmology, particularly for retinal and corneal diseases, due to its anti-inflammatory, antioxidant, antibacterial, and anti-angiogenic properties. Its antibacterial activity could enhance treatment options for ocular surface infections, such as conjunctivitis or keratitis, by directly combating bacterial pathogens. However, challenges related to bioavailability and solubility need to be overcome through advanced drug delivery systems like nanoparticles, niosomes, and cyclodextrin complexes.

Curcumin’s therapeutic value lies in its pleiotropic effects, including anti-inflammatory, antioxidant, and anti-angiogenic activities. In comparison to traditional treatments, curcumin offers a multi-targeted approach that may complement or enhance existing therapies. For example, its ability to prevent inflammation and oxidative damage positions it as a potential adjunct to anti-VEGF treatments for conditions like age-related macular degeneration. However, its clinical application is limited by poor bioavailability, necessitating further research to establish its clinical effectiveness relative to conventional treatments.

In conclusion, curcumin holds promising therapeutic potential for ophthalmology, but further studies, especially clinical trials, are required to confirm its clinical efficacy and overcome existing limitations. The continued exploration of innovative delivery systems will be key to unlocking its full therapeutic potential.

## Data Availability

Data are unavailable due to privacy.
